# A New Approach to Implant Stability Using a Flexible Synthetic Silicate-Additive Beta-Tricalcium Phosphate-Poly(D,L-lactide-*co*-caprolactone) Bone Graft: An In Vitro Study

**DOI:** 10.3390/polym16081101

**Published:** 2024-04-15

**Authors:** Zeynep Dilan Orhan, Levent Ciğerim

**Affiliations:** Department of Oral and Maxillofacial Surgery, Faculty of Dentistry, Van Yuzuncu Yil University, 65090 Van, Turkey; zeynepdilanorhan@yyu.edu.tr

**Keywords:** bone model, dental implant, implant stability, bone graft, flexible bone graft

## Abstract

The aim of this study was to evaluate the use of a flexible synthetic polymer bone graft to provide implant stability during implant placement in a dense cortical bone model. In the control group (Group 1), sockets were prepared on polyurethane blocks according to the standard implant socket drilling protocol; both oversizing and deepening were applied in Group 2; and only oversizing was applied in Group 3. In Groups 2 and 3, flexible synthetic polymer bone grafts were placed in the sockets prior to implant placement. The implants were placed at the bone level in all groups. The highest torque value obtained was recorded as the insertion torque. In this study, 75 implant sites were included across three groups. The torque values of the implants in the control group were significantly higher than those of the implants with the oversized and deepened sockets and the oversized-only sockets (*p* < 0.05; *p* < 0.01). The torque values of the implants with the oversized and deepened sockets were significantly higher than those of the implants with the oversized-only sockets (*p* < 0.01). In this study, a flexible synthetic polymer bone graft was shown to be effective in achieving implant stability in the management of implants where there has been a loss of primary stability.

## 1. Introduction

Currently, dental implants for the treatment of missing teeth are a routine option, and many factors affect their use as a treatment [1]. One of the most important factors for successful implant osseointegration is ensuring primary stability during implant placement [2]. Primary stability is the biometric stability achieved immediately after implant placement, when the implant is inserted into the designed open socket, and it consists of the mechanical attachment of the implant to the bone [3].

Osseointegration occurs without the need for movement in the bone socket after implant placement by inducing osteoblast proliferation and differentiation and inhibiting fibrous tissue ingrowth and encapsulation. Factors affecting primary stability include implant size, implant surface morphology and design, bone quality/quantity, the surgical technique used, and surgeon experience [1,3].

The assessment of primary stability at the site of implantation is a valid prognostic factor for successful osseointegration. A non-invasive clinical method, the insertion torque (IT) test, is commonly used to quantify the primary stability of implants [4]. It is a parameter that measures the frictional resistance encountered by the fixture as it rotates about its axis while advancing apically. The maximum IT is expressed in Newton centimeters (Ncm) and is predictive of the primary and secondary stability of an implant [5]. There is no consensus on the minimum IT required to achieve osseointegration. However, to achieve the “ideal” primary stability at the time of implant placement, oral surgeons generally recommend an IT of less than 55 Ncm [6,7]. It has been shown that IT values in this range prevent detrimental micromovement under implant loading, therefore promoting osseointegration [8]. Evidence from clinical experience shows that there is a linear relationship between primary stability and implant IT [9,10]. High ITs are associated with the possible occurrence of bone overcompression and, therefore, bone necrosis, which could lead to the failure of osseointegration. In addition, changes and deformations may occur in the various structures of the implants due to overload or the irregular distribution of forces caused by a high IT [11,12].

Local differences in bone anatomy and morphology may explain the differences in implant osseointegration results. The cortical-to-trabecular bone ratio is higher in the mandible than in the maxilla. Research suggests that implant survival is longer in the mandible than in the maxilla because the primary stability of the implant has been shown to be lower in low-density bone than in high-density bone [13,14]. Crestal cortical thickness and medial cancellous bone density and their relative distribution at the implant site determine the quantity and quality of host bone. Inadequate bone quantity and density are the major risk factors for implant failure because they are associated with excessive bone resorption and impaired healing [15]. Several studies show an association between increased failure rates and implant placement in D4 bones. In contrast, implants placed in D1–D3 bone are associated with good osseointegration. It is important to consider bone quality when planning implant placement, surgical procedures, healing time, and implant loading [16,17,18].

Synthetic polymers are almost as diverse as those found in nature, and new polymers have been quickly introduced into the medical field, such as polyester/polyamide synthetic sutures [19,20]. There are also functional types of polymers that have been developed for biomedical applications such as drug delivery devices, vascular stents, sutures, thrombectomy devices, aneurysm or ductus arteriosus closure, orthodontic therapy, and wound dressings [21,22,23]. The relative degradation rates and erosion mechanisms of hydrolytically degradable polymers are some of the key characteristics that significantly affect their ability to function as biomaterials [24,25,26]. In some cases, the degradation rates are highly modifiable by polymer chemistry, which offers significant flexibility in material properties. To determine how a degradable polymeric biomaterial will erode, degradation rates are combined with other factors such as water diffusion, monomer solubility and diffusion, and device geometry and size [26,27]. Poly(lactic acid) (PLA) and poly(gamma-caprolactone) (PCL) are hydrolytically degradable polymers that have been studied widely. Their advantages include biodegradability, non-toxic by-products, good biocompatibility, and the ability to allow drug penetration. PLA has high strength and low tenacity, whereas PCL has high tenacity and low strength. By combining the advantages of both, poly(l-lactide-co-ε-caprolactone) (PLC) can provide improved strength and toughness [23,28,29].

Another biomedical use of polymers is to improve the physical properties of bone grafts. A recently manufactured and marketed flexible synthetic polymer bone graft (FSPBG) is a synthetic bone void filler composed of beta-tricalcium phosphate (B-TCP) granules and the resorbable polymer poly[(D,L-lactide-*co*-caprolactone)]. FSPBG is a flexible, osteoconductive, three-dimensional composite material with excellent properties that conforms to the implant site, allowing site-specific placement [30,31,32]. One of the components of FSPBG is β-TCP, a synthetic bioceramic material widely used in the medical field. It is a biocompatible alloplastic bone graft material that is resorbable and has osteoconductive properties. It has been demonstrated that it is totally resorbed and replaced by new bone within 6–18 months [33]. The addition of silicate to beta-tricalcium phosphate has been shown to impart osteoinductive properties to the graft. The use of silicate as a substitute significantly increased the amount of bone that was formed and the amount of bone that was bonded to the surface of the implant [32,34].

Even if careful pre-operative planning is carried out prior to dental implant treatment and all conditions for dental implant placement are considered optimal, problems relating to local factors and the preparation of the surgical site may affect the success of the treatment [35]. If adequate stability is not provided during implant placement, then a micromovement may emerge, a fibrous tissue capsule may form, and the healing process may be disrupted, resulting in implant mobility and subsequent implant failure [2]. Routinely, the primary stability lost during implant placement is regained by replacing the implant with a wider implant; however, there is no established protocol in the literature for the management of this complication [36]. No studies in the literature have investigated achieving implant stability in implants where primary stability was not achieved at the time of implant placement. FSPBG is structurally flexible and resistant to disintegration and fragmentation under load. For this reason, FSPBG placed in the implant socket could work by filling the gap between the implant groove and the bone at any part of the implant and by creating a physical barrier to implant rotation by being compressed into this region. The aim of this study was to evaluate the use of FSPBG to achieve implant stability at zero insertion torque during implant placement in a dense cortical bone model.

## 2. Materials and Methods

**Figure 1:**
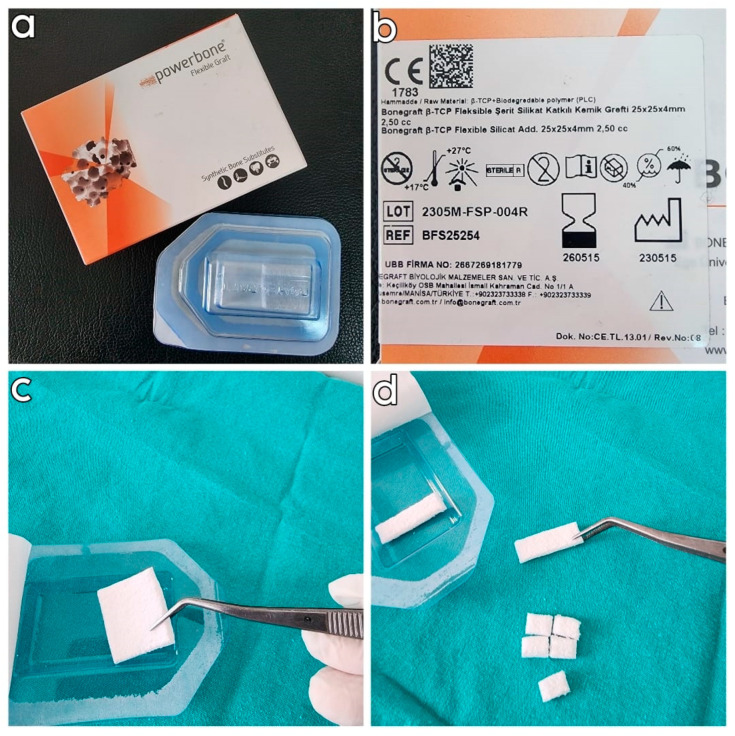
(**a**,**b**) The FSPBG used in this study had a size of 25 × 25 × 4 mm, which was used to produce 4 × 5 × 4 and 8 × 5 × 4 mm FGPBGs; (**c**) product characteristics; and (**d**) preparation of 8 × 5 × 4 mm and 4 × 5 × 4 mm FGPBGs.

This single-blind in vitro study was conducted at the Department of Oral and Maxillofacial Surgery, Faculty of Dentistry, Van Yüzüncü Yıl University, in January 2024. Polyurethane blocks measuring 25 × 10 × 2.5 cm with a density of 0.96 g/cm^3^ (60 per cubic foot (pcf) from PURYAP Construction Chemicals and Machinery Industry Trade. Co., Ltd., İstanbul, Turkey) were used to simulate the dense D1 bone model. A FSPBG (Bonegraft β-TCP Flexible Strip Silicate Add. Bone Graft, 25 × 25 × 4 mm, İzmir, Turkey) was used in the study groups (Figure 1). The FSPBGs used in this study were fabricated as follows. First, β-TCP and silicate were mixed in defined proportions (0.8% and 1%) and added to a 12.5% poly(D,L-lactide-co-caprolactone) polymer solution. A defined amount of porogen in a 1:1 ratio of 100–250 μm and 250–500 μm sizes was then incorporated into the mixture to impart a porous structure. The material was thoroughly mixed in the beaker and then poured into teflon-coated molds. The flexible bone graft was kept in distilled water in a shaking water bath to remove the porogen and obtain a porous structure in the material. The 8 × 5 × 4 mm and 4 × 5 × 4 mm FSPBGs were prepared from manufactured synthetic blocks with dimensions of 25 × 25 × 4 mm. The dimensions of the FSPBGs were controlled to ensure consistency. This study used 4.3 × 8 mm tapered platform-switch dental implants (Medisolaris Venuscon Implant, İzmir, Turkey) with an resorbable blasting media (RBM) surface. According to the sample size calculation made according to the reference article, it was determined that the minimum number of implant sockets in the study groups should be 24 (power= 99%; d = 1.494; α = 0.01) [37].

In the design of this in vitro study, oversized implant sockets were created on D1 bone density polyurethane plates in which the insertion torque value of the intended implant would be zero, in order to create a setting in which the implant had no primary stability. In this way, this study was originally planned to have 4 groups: 1 control group and 3 study groups, with a total of 100 implant sockets. As there was no parameter to determine the size of the FSPBG to be used in the sockets, we decided to use 2 different sizes of FSPBG that would not exceed the socket volume. In each group, implant sites were created according to a specific standard protocol. In all implant sites in the groups, 4.3 × 8 mm implants were placed at the bone level. To ensure that the investigators were blind in this study, all sockets were created by a surgeon outside this study according to the protocol specified for the groups, and only the assistants knew which sockets belonged to which group.

### 2.1. Drilling Protocol

All of the implant sockets in the groups were created by a single oral and maxillofacial surgeon who was outside of this study, specialized in their field, and actively performed implants at the time of their involvement. The sockets were created using a physiodispenser (Straumann Surgical Motor Pro, NSK Nakanishi Inc., Kanuma, Tochigi, Japan) and a 20:1 reduction implant handpiece (NSK S-Max SG20, Tochigi, Japan) at 700 revolutions per minute (rpm) and 35 torque using a standardized protocol. Again, as is standard protocol during implant placement, the implant placement process was completed by starting with 5 torques at 30 rpm and increasing the torque by 5 torques at the last and highest torque value when the implant was placed in the socket at bone level. The highest torque value obtained at this point was recorded as the IT.

### 2.2. Study Groups

**Figure 2:**
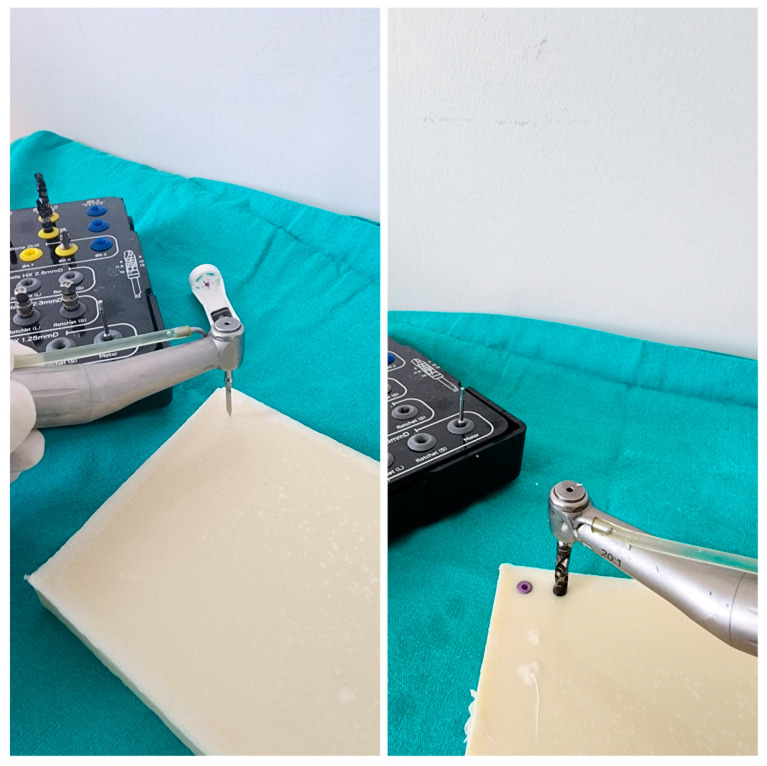
The application of the FSPBGs and implant placement after the preparation of the normal-sized sockets for the 4.3 × 8 mm diameter implant in the control group is shown.

The procedure used for Group 1 (control group) was as follows: The implant sockets were prepared using the standard 4.3 × 8 mm implant drilling protocol (the socket width was 3.8 mm in the neck region, 3.4 mm apically, and the socket length was 8 mm). The implants were placed at bone level (Figure 2). 

**Figure 3:**
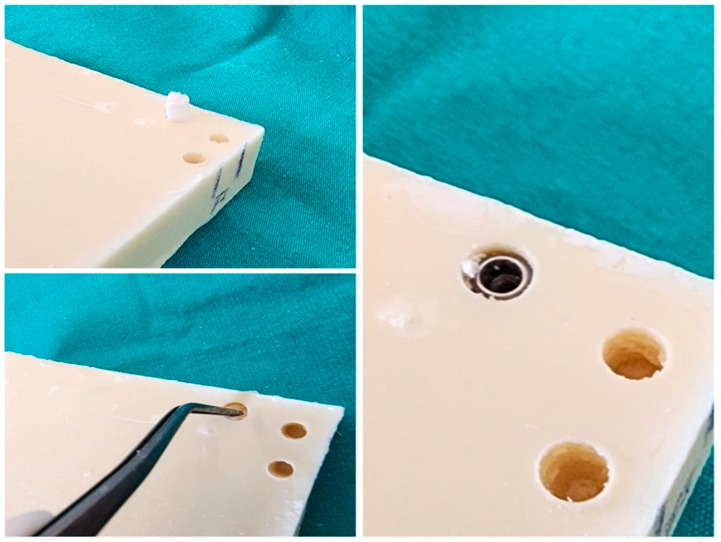
The application of the FSPBG and placement of the implant after preparation of the oversized sockets is shown.

The procedure used for Group 2 was as follows: The implant sockets were prepared using the standard drilling protocol for a 5.0 × 8 mm implant. The final drill of a 4.3 mm implant was then used to reach a depth of 10 mm (the socket width was 4.4 mm in the neck region, 3.8 mm apically, and the socket length was 10 mm). An 8 × 5 × 4 mm FSPBG was placed in the sockets. The implants were placed at bone level (Figure 3).

The procedure used for Group 3 was as follows: The implant sockets were prepared according to the standard drilling protocol for a 5.0 × 8 mm implant (the socket width was 4.4 mm in the neck region, 3.8 mm apically, and the socket length was 8 mm). Then, 4 × 5 × 4 mm FSPBGs were placed in the sockets. The implants were placed at bone level (Figure 3).

For Groups 2 and 3, the FSPBGs were kept in saline for 5 min prior to insertion into the sockets. No degradation or macroscopic changes in the material were observed during this time.

### 2.3. Statistical Analysis

To evaluate the results obtained in the study, SPSS 26.0.0 was used for statistical analyses. To evaluate the study data, quantitative variables were presented using mean, standard deviation, median values, minimum values, and maximum values, and qualitative variables were presented using descriptive statistical methods such as frequency and percentage. The Shapiro–Wilks test and box plots were used to assess the suitability of the data in terms of normal distribution. The Student’s *t*-test was used to evaluate two groups with normal distributions, a one-way ANOVA test was used to compare three or more groups, and the Bonferroni test was used to determine the group causing the difference. The results were evaluated using a 95% confidence interval and significance at the *p* < 0.05 level.

## 3. Results

When the socket groups used in this study were examined, 33.3% (n = 25) were controls, 33.3% (n = 25) were oversized and deepened, and 33.3% (n = 25) were oversized only. The IT values ranged between 10 and 65 Nm, with an average of 39.53 ± 13.46 Nm (Table 1).

A statistically significant difference was found between the IT values according to the groups (*p* = 0.001; *p* < 0.01). As a result of the pairwise comparisons made to determine the source of the difference, the IT values of the sockets in the control group were found to be significantly higher than those in the oversized and deepened sockets (*p* = 0.048; *p* < 0.05). The IT values of the sockets in the control group were significantly higher than those in the oversized-only sockets (*p* = 0.001; *p* < 0.01). The torque values of the oversized and deepened sockets were significantly higher than those of the oversized-only sockets (*p* = 0.001; *p* < 0.01) (Table 2).

The IT values of the sockets in the control group were statistically significantly higher than those of the oversized sockets (*p* = 0.001; *p* < 0.01), (Table 3).

## 4. Discussion

The increased use of dental implants in recent years has naturally led to an increase in the number of implant-related problems. Dental implant complications include infection, fracture of the jaw bone, damage to surrounding anatomical structures, bleeding, loss of hard or soft tissue in the peri-implant area, devitalization of the adjacent tooth, aspiration of the implant, fracture of the implant, loosening/fracture of the abutment screw, and loss of osseointegration of the implant. Although the cause of loss of osseointegration of the implant cannot be accurately predicted, it is generally believed to be due to the micromovement of the implant during the healing process. The primary stability of the implant is assessed using various methods such as maximum IT, implant stability quotient, and removal torque. To restore primary stability to an implant that has lost stability during implantation, it is replaced with a larger implant if there is sufficient surrounding tissue or if the defect area is grafted and the implant is reimplanted [38,39,40].

Today, scientific advances and government guidelines have led to a reduction in the use of all animal models in dental implant research. New development strategies are gradually replacing some in vivo experiments with in vitro or biomaterial approaches [41]. In this context, polyurethane models can be used as an alternative to human bone. The homogeneity and regularity of this model makes it an ideal material for comparative testing of bone screws and other medical instruments [42]. Looking at dental implant-related studies using polyurethane models, Di Stefano et al. compared methods for measuring primary implant stability on spongy bone-like polyurethane blocks of different densities and found that the IT–depth curve integral provides a reliable method for measuring primary stability [38]. Comuzzi et al. compared polyurethane blocks of different densities and reported that implant insertion and removal torques increased with increasing density [43]. Similarly, Stoilov et al. found that polyurethane blocks used in relation to different densities affected the ITs of the implants, with higher ITs being achieved with higher densities [44]. According to these studies, the use of polyurethane blocks for comparison between the ITs obtained from different sockets is considered appropriate. For this reason, a dense D1 bone-like polyurethane block (60 pcf) was used in this study, and as a result of the trials, it was observed both that the surgeon had a bone-like feeling when opening the socket and that consistent results were achieved after implant placement.

When analyzing the relationship between implant stability and the design of the implant socket, Haw-Ming Huang et al. prepared implant sockets in three different widths using rabbits: standard and narrower and wider than is standard. Their study discovered that the highest stability was found in undersized sockets. Histological examinations showed similar results in bone implant contact at the end of the second month [45]. In a study using the proximal tibia of sheep, Yurttan et al. used 4 mm diameter implants; one group had a 3.5 mm diameter socket and the other group had an enlarged socket with a 4.2 mm diameter socket. Primary stability could only be measured in the control group; resonance frequency analysis could not be performed in the enlarged socket. At the end of 3 months, similar removal torque values were found in the groups with and without primary stability [46]. In their study on rabbits, Cohen et al. placed 3.75 mm diameter implants in one group and 3.55 mm diameter implants in the other group, in sockets drilled to a diameter of 3.65 mm. Upon histological examination, they found similar bone-to-implant contact between the small- and large-diameter socket groups [47]. In contrast to these studies, in a study on the posterior maxilla, Seleem et al. made a socket to fit the implant in one group as recommended by the implant manufacturer, and in the other group, they made a socket 0.2 mm wider than the implant. At the end of the third month, the implant stability score was higher in the widened socket group than in the control group [48]. In their study on rat tibiae, Dündar et al. placed implants with primary stability in one group and implants without primary stability in the other. They reported that better osseointegration was achieved in the primary-stabilized group compared to the implants without primary stability [49]. The above studies have shown that osteointegration can be achieved in the long term in the absence of implant stability, but the rate of reintegration is higher for implants with initial stability. In addition, studies showing no difference in long-term osteointegration have shown that implants should have low initial stability. In this study, applying FSPBGs to the sockets during implant placement was shown to be effective in providing initial implant stability in implants without initial primary stability. The highest ITs were achieved in the control group with the sockets prepared according to the manufacturer’s instructions. When the two groups of oversized sockets were compared, it was found that the oversized and deepened sockets achieved higher torque values. This suggests that in the event of the loss of primary stability during implant placement, re-implantation by deepening the implant socket and applying FSPBGs to the socket will provide a more successful result if the surrounding anatomy allows. Again, in cases where socket deepening is not possible and implant stability is lost due to socket widening, we have shown that implant stability can be achieved by placing an FSPBG in the socket. No study was found in the literature that ensured the stability of implants without IT during implantation, and this study was the first in the literature to do so. The limitations of this study were as follows. It was observed that the FSPBG placed in the sockets in the study groups were placed apically during implant placement and very few of them were present laterally to the implant. Therefore, despite the primary stability of the implants, it is possible that the resistance to lateral forces is low in the initial phase. When determining the size of the grafts applied to the sockets, they were prepared to be close to the size of the socket. No other parameters were used. Although we have shown that an FSPBG is effective in mechanically compressing the implant in vitro, it is not known whether it will provide the same result in clinical settings. It has not been evaluated whether the primary stability achieved with the use of FSPBG in the oversized and deepened group can be achieved by simply placing the implant without the use of a graft for the same socket model. This study was designed to measure the removal torque of the implants in addition to the insertion torque. However, it was found that the lateral forces generated during removal due to the wide implant sockets negatively affected the measurements, and the evaluation of removal torque was canceled due to the lack of standardization of this factor. Today, implant surgery continues to be an increasing trend and we believe that this study will guide future studies to evaluate the clinical efficacy of FSPBG in late, early and immediate implant cases, particularly in immediate implant cases where problems related to the failure to achieve insertion torque are experienced, with this study supporting the use of FSPBG in this indication under in vitro conditions. This in vitro study demonstrated the use of FSPBG as a suitable material for implant stability due to its flexible structure, strength under load, and non-dispersibility. FSPBG is already commercially available and inexpensive. When FSPBG is evaluated in terms of the clinical studies to be carried out in this area, it has been introduced to the literature by highlighting the physical properties that make it preferable in material selection. Clinicians are recommended to use FSPBG in their clinical practice for this indication. Future case reports, case series, and clinical trials are needed to clinically prove the efficacy of FSPBG used with similar and/or different protocols as in this study to achieve implant stability. Again, for implants with zero insertion torque, FSPGB was found to increase and achieve primary stability of the implants when applied at low speed (10 rpm), starting from a low torque value (5 rpm) with a protocol of gradually increasing the torque value. In addition, in suitable cases where primary implant stability was zero, socket deepening was found to be an additional method to further increase implant stability. These results support that FSPBG, due to its physical properties, can be used to achieve primary stability in cases of zero or low implant stability with an appropriate placement protocol. Further studies are needed to support these results clinically.

## 5. Conclusions

In conclusion, this study was the first to evaluate the use of an FSPBG to achieve primary stability in cases of the loss of implant stability. The application of an FSPBG to oversized implant sockets was found to be successful in increasing implant stability; in addition, deepening the socket was found to further increase primary stability. Preventing implant failure due to zero insertion torque with FSPBG will reduce costs and the risk of complications for both physicians and patients. Further clinical studies are required to confirm the results of this in vitro study.

## Figures and Tables

**Table 1:** Distribution of implant socket numbers in groups and IT values.

		n (%)
	Group 1 (Control)	25 (33.3)
	Group 2 (Oversized and Deepened)	25 (33.3)
	Group 3 (Oversized only)	25 (33.3)
Insertion	Mean ± Standard deviation	39.53 ± 13.46
Torque Values (Nm)	Median (Min − Max)	40 (10–65)

**Table 2:** Comparison of the IT values by group.

	Torque Values (Nm)		^a^ *p*
	Mean ± Sd	Median (Min−Max)	
Group 1 (Control)	49.60 ± 7.63	50 (40–65)	0.001 *
Group 2 (Oversized and Deepened)	43.60 ± 7.84	45 (30–60)	
Group 3 (Oversized only)	25.40 ± 10.4	25 (10–50)	

^a^—One-Way ANOVA Test and Bonferroni Test, *—*p* < 0.01.

**Table 3:** Comparison of the IT values between the control and oversized groups.

	Torque Values (Nm)		^b^ *p*
	Mean ± Sd	Median (Min−Max)	
Control	49.60 ± 7.63	50 (40–65)	0.001 *
Oversized Groups	34.50 ± 12.95	35 (10–60)	

^b^—Student *t*-Test, *—*p* < 0.01.

## Data Availability

Data are contained within the article.
